# Methylation‐associated miR‐193b silencing activates master drivers of aggressive prostate cancer

**DOI:** 10.1002/1878-0261.12536

**Published:** 2019-07-19

**Authors:** Ying Z. Mazzu, Yuki Yoshikawa, Subhiksha Nandakumar, Goutam Chakraborty, Joshua Armenia, Lina E. Jehane, Gwo‐Shu Mary Lee, Philip W. Kantoff

**Affiliations:** ^1^ Department of Medicine Memorial Sloan Kettering Cancer Center New York New York USA; ^2^ Center for Molecular Oncology Memorial Sloan Kettering Cancer Center New York New York USA; ^3^ Department of Medical Oncology Dana‐Farber Cancer Institute Boston Massachusetts USA

**Keywords:** miR‐193b, FOXM1, RRM2, prostate cancer, DNA methylation

## Abstract

Epigenetic silencing of miRNA is a primary mechanism of aberrant miRNA expression in cancer, and hypermethylation of miRNA promoters has been reported to contribute to prostate cancer initiation and progression. Recent data have shown that the miR‐193b promoter is hypermethylated in prostate cancer compared with normal tissue, but studies assessing its functional significance have not been performed. We aimed to elucidate the function of miR‐193b and identify its critical targets in prostate cancer. We observed an inverse correlation between miR‐193b level and methylation of its promoter in The Cancer Genome Atlas (TCGA) cohort. Overexpression of miR‐193b in prostate cancer cell lines inhibited invasion and induced apoptosis. We found that a majority of the top 150 genes downregulated when miR‐193b was overexpressed in liposarcoma are overexpressed in metastatic prostate cancer and that 41 miR‐193b target genes overlapped with the 86 genes in the aggressive prostate cancer subtype 1 (PCS1) signature. Overexpression of miR‐193b led to the inhibition of the majority of the 41 genes in prostate cancer cell lines. High expression of the 41 genes was correlated with recurrence of prostate cancer. Knockdown of miR‐193b targets *FOXM1* and *RRM2* in prostate cancer cells phenocopied overexpression of miR‐193b. Dual treatment with DNA methyltransferase (DNMT) and histone deacetylase (HDAC) inhibitors decreased miR‐193b promoter methylation and restored inhibition of *FOXM1* and *RRM2*. Our data suggest that silencing of miR‐193b through promoter methylation may release the inhibition of PCS1 genes, contributing to prostate cancer progression and suggesting a possible therapeutic strategy for aggressive prostate cancer.

**Abbreviations**
5‐Aza‐dC: 5‐Aza‐2’‐deoxycytidine
DNMT: DNA methyltransferase
DNMTi: DNA methyltransferase inhibitor
GSEA: gene set enrichment analysis
HDAC: histone deacetylase
HDACi: histone deacetylase inhibitor
miRNA: small noncoding microRNA
MSP: methylation‐specific PCR
PC: prostate cancer
PCS1: prostate cancer subtype 1
TCGA: The Cancer Genome Atlas

## 1 Introduction

Epigenetic mechanisms of gene expression regulation, including DNA methylation, histone modifications, and small noncoding microRNA (miRNA), play critical roles in cancer initiation and progression (Suva *et al.*, 2013). Aberrant epigenetic regulation can cause altered gene expression and malignant cellular transformation. miRNA regulate a wide range of biological processes by repressing the expression of target genes (Krol *et al.*, 2010), and their dysregulation can contribute to the development and progression of cancer. Growing evidence has demonstrated that miRNA can be epigenetically silenced in various cancers (Suzuki *et al.*, 2012), including by methylation at CpG regions within miRNA promoters (Weber *et al.*, 2007).

Epigenetic alterations, including perturbed DNA methylation at the promoters of critical genes (*AR*, *GSTP1*, and *PTEN*) and altered histone modifications, have been reported to contribute to prostate cancer (PC) progression (Nelson *et al.*, 2009; Ruggero *et al.*, 2018; Valdes‐Mora and Clark, 2015). Depending on the function of their target genes, miRNA have been reported to function as both tumor suppressors and oncogenes in PC (Kanwal *et al.*, 2017). A recent meta‐analysis of published miRNA profiling studies in PC demonstrated that dysregulation of miRNA is highly correlated with PC onset, progression, and metastasis, suggesting that miRNA could serve as potential biomarkers in PC (Song *et al.*, 2018). Aberrant methylation of miR‐193b has been reported in liposarcoma, lymphoma, and cervical and gastric cancers (Du *et al.*, 2012; Jimenez‐Wences *et al.*, 2016; Mazzu *et al.*, 2017). In liposarcoma, miR‐193b has been shown to function as a tumor suppressor (Mazzu *et al.*, 2017). Several studies have revealed that the miR‐193b promoter is hypermethylated in prostate tumors and in the urine of patients with PC (Moreira‐Barbosa *et al.*, 2018; Rauhala *et al.*, 2010; Torres‐Ferreira *et al.*, 2017), but our understanding of miR‐193b function and its critical targets in PC remains limited.

In this study, we demonstrated that miR‐193b has tumor suppressive functions in PC cells. Furthermore, we discovered a network of 41 miR‐193b target genes that are activated in aggressive PC. We validated that 2 of these genes—*FOXM1* and *RRM2*—are direct targets of miR‐193b and that aberrant upregulation of both genes is associated with aggressive PC. Finally, we demonstrated that dual treatment with DNMT and HDAC inhibitors restored miR‐193b expression and inhibitory function.

## 2 Materials and methods

### 2.1 Cell culture

Normal human prostate (RWPE‐1 and PZ‐HPV‐7) and PC cells (LNCaP, 22Rv1, DU145, and PC‐3) were purchased from ATCC (Manassas, VA, USA). E006AA cells were provided by John T. Isaacs (The Johns Hopkins University School of Medicine, Baltimore, MD, USA). The LAPC4 cell line was provided by Charles Sawyers (Memorial Sloan Kettering Cancer Center, New York, NY, USA), and C4‐2 cells were obtained from VitroMed (Burlington, NC, USA). All cells were maintained in media with 10% fetal bovine serum (FBS) supplemented with 2 mm of L‐glutamine and penicillin/streptomycin (100 U·mL^−1^) at 37 °C in 5% CO_2_. Cells were authenticated by human short tandem repeat profiling at the Memorial Sloan Kettering Cancer Center Integrated Genomics Operation.

### 2.2 Reagents and plasmids

miRNA (catalog numbers 4464058 [nonspecific] and 4464066 [miR‐193b]) and anti‐miRNA (catalog numbers 4464076 [nonspecific] and 4464084 [anti‐miR‐193b]) were purchased from Ambion (Foster City, CA, USA). SMARTpool siRNA for *FOXM1* and *RRM2* were purchased from Dharmacon (Lafayette, CO, USA). 5‐Aza‐dC and mocetinostat were purchased from Selleck Chemicals (Houston, TX, USA). Wild‐type and mutant *FOXM1* and *RRM2* 3’UTR reporters were purchased from Switchgear Genomics (Carlsbad, CA, USA). The mutated oligonucleotide sequences are shown in Table S1.

### 2.3 Transfection of miRNA and siRNA and luciferase assays

miRNA and anti‐miRNA were transfected at a concentration of 50 nm with Oligofectamine (Thermo Fisher Scientific, Waltham, MA, USA) into subconfluent (50%) cells. Cells were harvested 72 h after transfection for protein and mRNA analysis.

SMARTpool siRNA were transfected with RNAiMAX (Thermo Fisher Scientific). Cells were harvested 48 or 72 h after transfection for protein and mRNA analysis. Efficiency of knockdown and overexpression was verified by qPCR and western blot.

For 3’UTR luciferase reporter assays, miRNA or siRNA were co‐transfected with 500 ng of reporter using Lipofectamine 2000 (Thermo Fisher Scientific). Cells were harvested 48 h after transfection for luciferase assays. Luciferase activity assays were performed using LightSwitch Luciferase Assay Kit (Switchgear Genomics) according to the manufacturer’s instructions.

### 2.4 RNA analysis and immunoblotting

Total RNA was extracted and analyzed as previously described (Zhang *et al.*, 2011). RNA was reverse transcribed using the qScript cDNA synthesis kit and qScript microRNA cDNA synthesis kit (Quanta Bioscience, Beverly, MA, USA). TaqMan gene expression assays (Thermo Fisher Scientific) were used for RRM2 and FOXM1 pathway gene expression. miRNA expression levels were detected using SYBR Green and miRNA‐specific primers (Thermo Fisher Scientific). Probe information and primer sequences are listed in Table S1. Expression of the 41 miR‐193b target genes (and 6 control genes) was validated with PrimePCR assays (Bio‐Rad, Hercules, CA, USA). Primer information is provided in Table S2. Expression levels were assessed by qPCR on an QuantStudio 5 Real‐Time PCR System (Thermo Fisher Scientific). Transcript levels were normalized to levels of GAPDH mRNA (for mRNA) or U6 snRNA (for miRNA).

Cells were lysed on ice in radioimmunoprecipitation assay (RIPA) buffer containing Halt Protease and Phosphatase Inhibitor Cocktail (Thermo Fisher Scientific). Protein concentration was determined by the Pierce Detergent Compatible Bradford Assay Kit (Thermo Fisher Scientific). Proteins were separated on 4–12% SDS/PAGE gels and transferred onto polyvinylidene difluoride (PVDF) membranes (MilliporeSigma, Burlington, MA, USA). Membranes were blocked with 5% nonfat milk and then incubated with primary antibodies. The sources of antibodies were as follows: FOXM1 (sc‐376471, 1 : 1000), RRM2 (sc‐81850, 1 : 1000), and beta‐actin (sc‐1615, 1 : 2500) antibodies were ordered from Santa Cruz Biotechnology (Dallas, TX, USA); antibodies for PARP (9542L, 1 : 2000), cleaved caspase‐3 (9661S, 1 : 1000), E‐cadherin (3195S, 1 : 1000), and N‐cadherin (13116S, 1 : 1000) were from Cell Signaling (Danvers, MA, USA). After incubation with secondary antibodies, the proteins of interest were detected with an ECL system (GE Healthcare, Chicago, IL, USA).

### 2.5 Cell viability and apoptosis assays

Cell viability was evaluated with the CellTiter‐Glo Luminescent Cell Viability Assay (Promega, Madison, WI, USA). At 72 h post‐transfection, apoptosis was quantified using the Muse Annexin V and Dead Cell Assay Kit (MilliporeSigma). Cells were cultured in 6‐well plates to 50% confluency, then transfected with miRNA or siRNA. After 72 h, cells were collected in media with 1% FBS, mixed with Muse Annexin V and Dead Cell Reagent, and analyzed using a Muse Cell Analyzer (MilliporeSigma).

### 2.6 Invasion assay

8 × 10^4^ 22Rv1 cells, 5 × 10^4^ PC‐3 cells, and 1.2 × 10^5^ LNCaP cells were plated in each well on the top of Matrigel invasion chambers (Fisher Scientific) in serum‐free media. Media supplemented with 10% FBS was used as the chemo‐attractant in the lower chamber. After 48 h (for PC‐3) or 72 h (for 22Rv1 and LNCaP), cells in the bottom chamber were fixed in methanol, stained with crystal violet, photographed, and counted under phase‐contrast microscopy.

### 2.7 Methylation‐specific PCR (MSP)

Genomic DNA was extracted from PC cell lines using the DNeasy Blood and Tissue Kit (Qiagen, Venlo, Netherlands). DNA methylation status was detected by MSP as previously described (Mazzu *et al.*, 2017). Briefly, bisulfite conversion of DNA was performed using the EpiTect Bisulfite Kit (Qiagen). CpGenome Universal Methylated DNA (MilliporeSigma) was used as a positive control. PCR was performed using the EpiTect MSP Kit (Qiagen) with the following cycling conditions: 95 °C for 10 min; 35 cycles of 94 °C for 15 s, 52 °C for 30 s, and 72 °C for 30 s; final extension at 72 °C for 10 min. MSP primers from a previous study were used (Mazzu *et al.*, 2017), and primer sequences are listed in Table S1.

### 2.8 Bioinformatic analysis of clinical cohorts

PC clinical cohorts are summarized in Table S3. Bioinformatic analysis of the various clinical cohorts was performed using data obtained from cBioPortal for Cancer Genomics (Gao *et al.*, 2013) and Oncomine (Rhodes *et al.*, 2004). The *z*‐score for each gene in 41‐gene list was generated based on the mRNA expression data from the primary PC samples in the Taylor cohort. An mRNA score was obtained by summing the *z*‐scores of all 41 genes. This generated a unique value for each sample in the cohort, which was used to sort samples into low, intermediate, and high expression quartiles. Kaplan–Meier survival curves were used to correlate mRNA score with disease‐free survival, and log‐rank tests were used to assess the significance of survival differences. Heatmaps were generated using r version 3.4.3 (https://www.R-project.org). Pathway analysis from RNA sequencing data was performed using GSEA (Subramanian *et al.*, 2005) and ToppGene (Chen *et al.*, 2009).

### 2.9 Statistical analysis

Statistical analysis was carried out with r version 3.4.3 or graphpad prism version 5.0 (GraphPad Software Inc., San Diego, CA, USA). All data were represented as mean ± standard error of the mean (SEM) from at least three independent experiments. Statistical analysis was performed with Student's *t*‐test (for comparisons of two groups) or one‐way ANOVA followed by Dunnett's test (for multiple group comparisons) where appropriate. Disease‐free survival was analyzed using Kaplan–Meier curves, and log‐rank tests were used to assess the significance of survival differences.

## 3 Results

### 3.1 The miR‐193b promoter is methylated in PC tumors and cell lines

**Figure 1:**
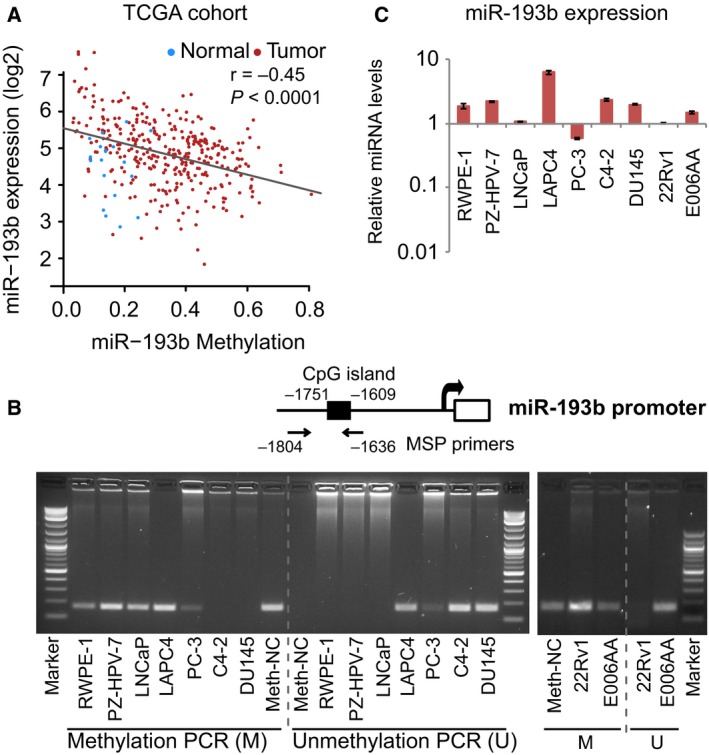
miR‐193b expression and promoter methylation in prostate cancer tissues and cell lines. (A) the correlation between miR‐193b expression level and promoter methylation status in normal (*n* = 19) and PC samples (*n* = 333) from TCGA cohort. miR‐193b promoter methylation level is measured in β‐value, with 0 being unmethylated and 1 being fully methylated. The *r* and *P* values shown are for the PC samples only. (B) methylation of the miR‐193b promoter was assessed by methylation‐specific PCR in 2 normal prostate cell lines (RWPE‐1 and PZ‐HPV‐7) and 7 PC cell lines (LNCaP, LAPC4, PC‐3, C4‐2, DU145, 22Rv1, and E006AA). Top, schematic of the miR‐193b promoter showing location of the CpG island and primers to assess methylation (MSP). Below, agarose gel showing the results of methylation and unmethylation PCRproducts. Methylation‐specific primers were used for methylated PCR, and unmethylated‐specific primers were used for unmethylation PCR. Methylated human genomic DNA (Meth‐NC) was used as a positive control for the methylation reaction. (C) miR‐193b expression in normal prostate and PC cell lines was assessed by qRT‐PCR. Expression values were normalized to the level of miR‐193b in 22Rv1 cells, which we assigned a value of 1. Values represent the mean ± standard error of the mean (SEM) of three independent experiments.

To validate the previously reported hypermethylation of the miR‐193b promoter in PC, we analyzed the correlation between miR‐193b level and methylation status of its promoter in a large (*n* = 333) PC clinical cohort (Cancer Genome Atlas Research Network, 2015). Methylation of the promoter was highly correlated with reduced miR‐193b expression in PC (*r* = −0.45, *P* < 0.0001) but not in normal prostate (*n* = 19; Fig. 1A). We assessed the expression of miR‐193b by quantitative reverse transcription PCR (qRT‐PCR) and the methylation status of the miR‐193b promoter by methylation‐specific PCR (MSP) in 7 human PC cell lines and 2 immortalized prostate epithelial cell lines (Fig. 1B,C). In 2 PC cell lines (LNCaP and 22Rv1), we observed complete methylation of the miR‐193b promoter and low levels of miR‐193b expression. The miR‐193b promoter was unmethylated in C4‐2 and DU145 cells, leading to moderate expression of miR‐193b. Three PC cell lines (LAPC4, PC‐3, and E006AA) exhibited heterogenous methylation of the miR‐193b promoter; expression of miR‐193b varied widely in these cell lines, with LAPC4 having the highest and PC‐3 having the lowest expression in all cell lines. Surprisingly, complete methylation of the miR‐193b promoter was observed in the 2 normal prostate epithelial cell lines. However, miR‐193b levels in these cell lines were still about twofold higher than in PC cell lines with methylated miR‐193b promoters (LNCaP and 22Rv1), indicating that there are additional mechanisms, such as those affecting miRNA stability, that regulate miR‐193b expression in normal prostate and PC cells. Altogether, we confirmed methylation of the miR‐193b promoter in PC tissues and cell lines.

### 3.2 Overexpression of miR‐193b decreases the viability of PC cells

**Figure 2:**
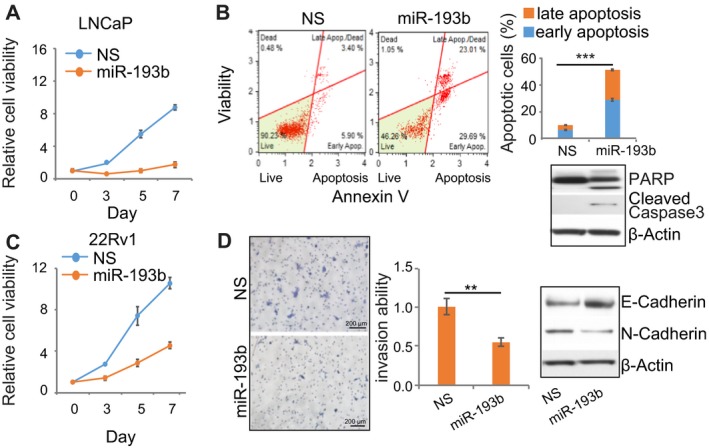
miR‐193b overexpression decreases the viability of PC cells. (A) decreased cell viability in LNCaP cells that overexpress miR‐193b. Cells were replated 1 day after transfection with miRNA, and cell viability was monitored by CellTiter‐Glo assay from Day 0 to Day 7. All values were normalized to the value of the nonspecific control miRNA (NS) on Day 0. (B) apoptosis levels in LNCaP cells that overexpress miR‐193b or NS miRNA. Apoptosis was detected 72 h after transfection. Left, FACS plots with percentages of cells in each quadrant shown. Top right, quantification of FACS results. Bottom right, apoptosis markers assessed by immunoblotting. (C) cell growth of 22Rv1 cells that overexpress miR‐193b or NS miRNA. Values were normalized to the value of NS control group on Day 0. (D) invasion of 22Rv1 cells overexpressing NS miRNA or miR‐193b. Left, representative images of results. Middle, quantification of invasion assay results. Right, markers of epithelial–mesenchymal transition were assessed by immunoblotting. Scale bar: 200 µm. Values represent the mean ± SEM of three independent experiments. Student's *t*‐test was used for *P* value calculation. **P < 0.01; ***P < 0.001.

To investigate the function of miR‐193b in PC, we overexpressed miR‐193b and control miRNA in PC cells. miR‐193b overexpression decreased the viability of one normal prostate and six PC cell lines (Fig. 2A,C; Fig. S1A). Levels of miR‐193b were increased over 100‐fold with ectopic expression (Fig. S1B). We used an inhibitor specific for miR‐193b (anti‐miR‐193b) to examine the effect of miR‐193b inhibition in LAPC4 cells, which had the highest levels of miR‐193b among the tested cell lines. Inhibition of miR‐193b induced a modest but significant increase in cell viability (Fig. S1C). Overexpression of miR‐193b led to high levels of apoptosis (> 50% of cells) and cleavage of PARP and caspase‐3 in LNCaP cells (Fig. 2B) but induced only low levels (< 10% of cells) of apoptosis in 22Rv1 cells (Fig. S1D). miR‐193b overexpression inhibited invasion by approximately 50% in both 22Rv1 (Fig. 2D) and PC‐3 cells (Fig. S1E) and resulted in upregulation of E‐cadherin and downregulation of N‐cadherin (Fig. 2D), suggestive of inhibited epithelial–mesenchymal transition.

### 3.3 Expression of 41 PCS1 signature genes is inhibited by overexpression of miR‐193b in PC cells

**Figure 3:**
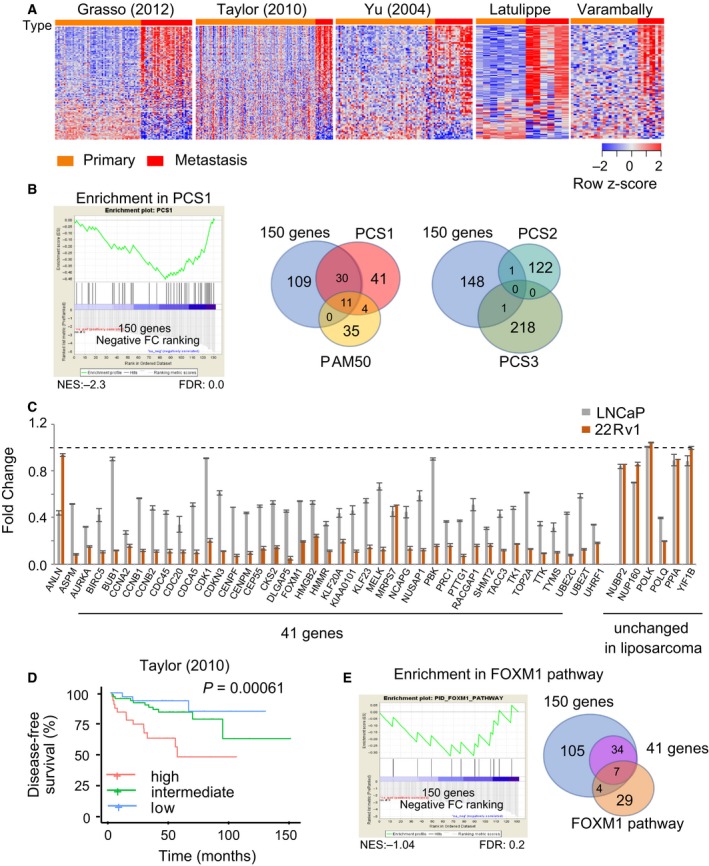
miR‐193b targets PCS1 genes to inhibit PC progression. (A) Expression of 150 miR‐193b‐inhibited genes in PC. The expression of the top 150 downregulated genes by miR‐193b in liposarcoma cells was assessed in 5 PC cohorts. Heatmaps were generated by supervised hierarchical clustering of primary (orange) and metastatic (red) PC samples. (B) Overlap between miR‐193b targets, PCS1 signature genes, and PAM50 genes. Left, GSEA plot of PCS1 signature gene enrichment in the 150 miR‐193b target genes. Right, Venn diagrams demonstrating the overlap between miR‐193b target genes and genes in the PCS1, PCS2, PCS3, and PAM50 signatures. (C) Expression of 41 genes was assessed after miR‐193b overexpression in PC cell lines. The expression of the 41 genes identified in (B) was evaluated by qRT‐PCR in LNCaP and 22Rv1 cells that overexpress miR‐193b. Expression levels were normalized to the control group (NS). Six genes unaffected by miR‐193b overexpression in liposarcoma cells were selected as negative control. (D) Association between expression of the 41‐gene panel and disease‐free survival of primary PC cases in the Taylor cohort. (E) Enrichment of miR‐193b‐regulated genes in *FOXM1* pathway. Left, GSEA plot of *FOXM1* pathway enrichment in miR‐193b target genes. Right, Venn diagram demonstrating the overlap between miR‐193b and *FOXM1* target genes. Values represent the mean ± SEM of three independent experiments. Disease‐free survival was analyzed using Kaplan–Meier curves, and log‐rank tests were used to assess the significance of survival differences.

To elucidate how silencing of miR‐193b contributes to PC progression, we sought to identify the targets of miR‐193b. Because of the conservation of miRNA targets (Krol *et al.*, 2010), we hypothesized that miR‐193b regulates the same network of genes in different cell types. We selected the top 150 downregulated genes (FC < −2.5, FDR < 0.01; GSE83690) when miR‐193b is overexpressed in liposarcoma cells (Mazzu *et al.*, 2017) and examined the expression of these genes in 5 PC cohorts. We found that a majority were upregulated in metastatic samples compared with primary samples (Fig. 3A). Recent analyses have described gene sets that can be used to predict PC prognosis: the PC subtypes (PCS1–3) and the PAM50 classifier (You *et al.*, 2016; Zhao *et al.*, 2017). We applied gene set enrichment analysis (GSEA) to determine whether these 150 genes were enriched in the PCS subtypes or the PAM50 classifier. We found a significant enrichment of the 150 miR‐193b‐inhibited genes with the 86 genes that are activated in the PCS1 subtype, the most aggressive of the three PC subtypes (NES: −2.3; FDR: 0.0; Fig. 3B). Of the 150 miR‐193b target genes, 41 genes are activated in the PCS1 subtype, but only 1 miR‐193b target gene is activated in the less aggressive PCS2 and PCS3 signatures (Fig. 3B). Eleven of the 41 miR‐193b target genes that are activated in the PCS1 subtype are also found in the PAM50 gene set.

We hypothesized that the 41 genes are miR‐193b‐regulated genes in PC. An *in silico* analysis using Toppgene (Chen *et al.*, 2009) found miR‐193b as the top candidate miRNA inhibiting these 41 genes, suggesting approximately 16 of the 41 genes could be direct targets of miR‐193b (Fig. S2A). We also overexpressed miR‐193b in PC cell lines and evaluated the expression of the 41 genes. In LNCaP cells overexpressing miR‐193b, the expression of 38 of the 41 genes was inhibited by more than 35% (Fig. 3C). In 22Rv1 cells, the expression of 39 of 41 genes was repressed by at least 75% (Fig. 3C). In PC‐3 cells, the expression of 38 of 41 genes was downregulated by 40% with miR‐193b overexpression (Fig. S2B). Differences in the extent of miR‐193b overexpression in the 3 cell lines may have contributed to the different degrees of target gene inhibition (Fig. S2C). We also selected 6 genes whose expression was not affected by miR‐193b at both the mRNA and protein level in liposarcoma cells (Mazzu *et al.*, 2017) and assessed whether their expression changed with miR‐193b overexpression in PC cells. Of these genes, only *POLQ* was differentially expressed with miR‐193b overexpression (Fig. 3C). Importantly, high expression of the 41 genes was correlated with decreased disease‐free survival in the Taylor cohort (*P* = 0.00061, Fig. 3D). Using gene ontology analysis and GSEA, we demonstrated that the 150 miR‐193b‐regulated genes were significantly enriched in the FOXM1 pathway and that 7 of the 41 genes are in the FOXM1 pathway (Fig. 3E). Pathway analysis of the 41 miR‐193b PCS1 genes confirmed enrichment in the FOXM1 pathway (Fig. S2D).

### 3.4 miR‐193b directly targets *FOXM1* in PC

**Figure 4:**
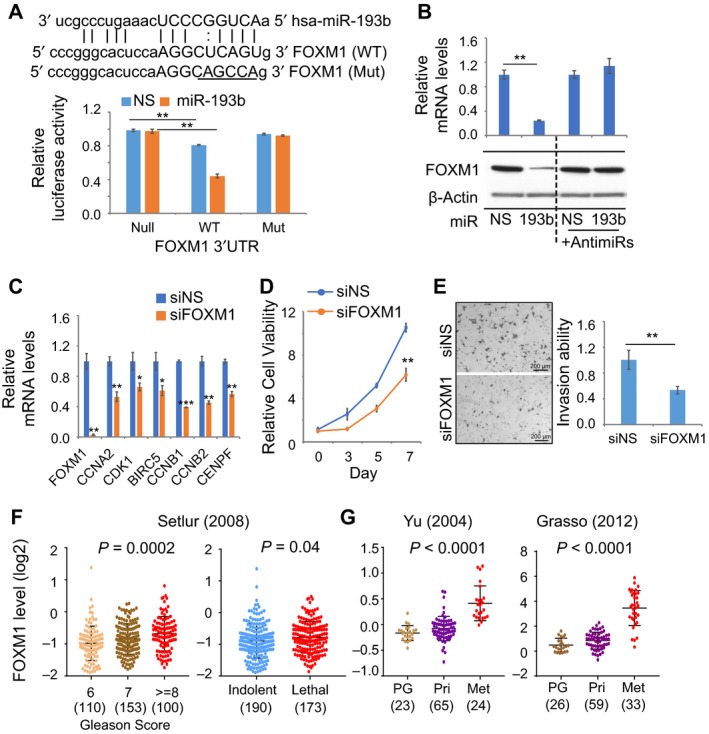
miR‐193b directly targets *FOXM1* in PC cells. (A) Inhibition of *FOXM1* 3’UTR activity by miR‐193b in 22Rv1 cells. Top, the wild‐type (WT) and mutant (Mut) seed regions of miR‐193b in the 3’UTR of *FOXM1* are shown. Underlined nucleotides in the seed region have been mutated in the mutant reporter. Bottom, luciferase activities were detected in three luciferase reporters containing no (Null), wild‐type, or mutated miR‐193b seed region in *FOXM1* 3’UTR with nonspecific (blue) or miR‐193b (orange) overexpression. Activities were normalized to null reporter values. (B) Inhibition of *FOXM1* expression by miR‐193b. miRNA were transfected with or without miRNA inhibitors (anti‐miR) in 22Rv1 cells. *FOXM1* expression was assessed at the mRNA and protein levels by qRT‐PCR and immunoblotting, respectively. Values were normalized to NS. (C) Decreased *FOXM1* target gene expression with knockdown of *FOXM1*. Expression of 7 *FOXM1* target genes was detected by qRT‐PCR in 22Rv1 cells transfected with NS or *FOXM1* siRNA. (D) Inhibition of cell viability by knockdown of *FOXM1*. Values were normalized to the NS control value at Day 0. (E) Inhibition of invasion by knockdown of *FOXM1*. Left, representative images of results. Right, quantification of results. Scale bar: 200 µm. (F) Correlation of *FOXM1* expression level with Gleason score (left) and lethality (right) in the Setlur cohort. The number of cases in each category is shown. (G) Association of *FOXM1* expression level with disease progression in the Yu (left) and Grasso (right) cohorts. The number of patients in each category is shown. PG, prostate gland; Pri, primary PC; Met, metastatic PC. Values represent the mean ± SEM of three independent experiments. Statistical analysis was performed with Student's *t*‐test (for comparisons of two groups) or one‐way ANOVA followed by Dunnett's test (for multiple group comparisons). *P < 0.05; **P < 0.01; ***P < 0.001.

We used a luciferase reporter assay to test our hypothesis that miR‐193b might directly target *FOXM1* and regulate the FOXM1 pathway. As compared to the control, the *FOXM1* 3’UTR luciferase reporter levels were 20% lower (Fig. 4A). Overexpression of miR‐193b led to a 50% reduction in the activity of the *FOXM1* 3’UTR reporter, and mutation of the miR‐193b seed region in the *FOXM1* 3’UTR restored reporter activity (Fig. 4A). Furthermore, overexpression of miR‐193b significantly inhibited FOXM1 expression at both the mRNA and protein level, which was blocked by anti‐miR‐193b (Fig. 4B). Altogether, these results demonstrate that miR‐193b directly binds to the *FOXM1* 3’UTR to inhibit expression in PC cells.

Seven of the 41 miR‐193b target genes are FOXM1 target genes (Fig. 3E); the expression of these genes was repressed by knockdown of *FOXM1* (Fig. 4C). Knockdown of *FOXM1* in 22Rv1 cells significantly inhibited cell viability and cell invasion, which was similar to phenotypes observed with miR‐193b overexpression (Fig. 4D,E). In contrast, knockdown of *FOXM1* in LNCaP cells, which have lower levels of FOXM1 than 22Rv1 cells (Fig S3A), modestly reduced cell viability but did not significantly inhibit cell invasion (Fig. S3B,C). Knockdown of *FOXM1* in both cell lines was confirmed by western blot (Fig. S3D). Because *FOXM1* is in the PCS1 signature and was reported as a master driver of PCS1 tumors (Ketola *et al.*, 2017), we analyzed the correlation between *FOXM1* upregulation and clinical outcomes in PC cohorts. In the Setlur cohort (also known as the Swedish Watchful Waiting cohort) of 363 primary PC samples (Setlur *et al.*, 2008), there was a significant correlation between increased *FOXM1* levels and higher Gleason score (*P* = 0.0002) and disease lethality (*P* = 0.04, Fig. 4F). In two cohorts with gene expression analysis of benign prostates and primary and metastatic PC cases (Grasso *et al.*, 2012; Yu *et al.*, 2004), *FOXM1* expression was significantly higher in metastatic cases (Fig. 4G). Altogether, this suggests that miR‐193b directly targets *FOXM1* to promote PC progression.

### 3.5 *RRM2* is a direct target of miR‐193b in PC cells

**Figure 5:**
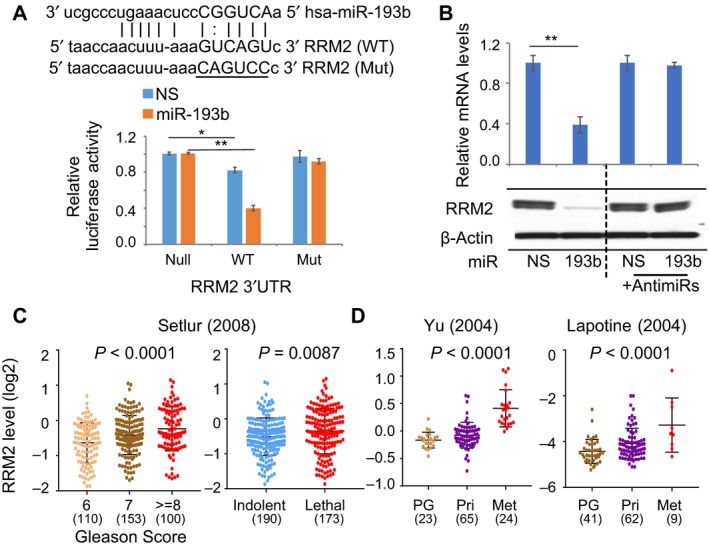
*RRM2* is the direct target of miR‐193b in PC cells. (A) Inhibition of *RRM2* 3’UTR activity by miR‐193b in LNCaP cells. Top, wild‐type (WT) and mutant (Mut) seed regions of miR‐193b in the 3’UTR of *RRM2* are shown. Bottom, luciferase activities were detected in three luciferase reporters containing no (Null), wild‐type, or mutated miR‐193b seed region in *RRM2* 3’UTR. Activities were normalized to null reporter values. (B) Inhibition of *RRM2* expression by miR‐193b. LNCaP cells were transfected with miRNA and miRNA inhibitors (anti‐miR), and *RRM2* expression was assessed at the mRNA and protein levels by qRT‐PCR and immunoblotting, respectively. Values were normalized against the NS values. (C) Correlation between *RRM2* expression level and Gleason score (left) and lethality (right) in the Setlur cohort. The number of cases in each category is shown. (D) Association of *RRM2* expression level with disease progression in the Yu (left) and Lapotine (right) cohorts. The number of cases in each category is shown. Values represent the mean ± SEM of three independent experiments. Statistical analysis was performed with Student's t‐test (for comparisons of two groups) or one‐way ANOVA followed by Dunnett's test (for multiple group comparisons). *P < 0.05; **P < 0.01.

miR‐193b overexpression induced distinct phenotypes in LNCaP and 22Rv1 cells (Fig. 2), and we demonstrated miR‐193b targets *FOXM1* in 22Rv1 cells to inhibit invasion (Fig. 4). However, knockdown of *FOXM1* had only modest effects in LNCaP cells (Fig. S3). To identify the miR‐193b target in LNCaP cells that regulates cell survival, we examined the top proteins with differential expression in liposarcoma cells that overexpress miR‐193b (Mazzu *et al.*, 2017). *RRM2*, which had the second highest reduction in protein expression, has been reported to be required for cancer cell survival due to its essential function in producing deoxyribonucleotide triphosphates (dNTPs; D’Angiolella *et al.*, 2012). We have previously shown that knockdown of *RRM2* leads to significant cell growth inhibition and induction of apoptosis in LNCaP cells (Mazzu *et al.*, 2019); miR‐193b overexpression results in the same phenotype (Fig. 2A, 2). *RRM2* is also 1 of 86 genes in the PCS1 signature. To determine whether *RRM2* is a direct target of miR‐193b in PC, we used luciferase reporter assays in LNCaP cells. Activity of the wild‐type *RRM2* 3’UTR reporter was approximately 20% lower than the control reporter, and exogenous miR‐193b induced a 60% reduction in activity (Fig. 5A). Mutation of the seed regions in the *RRM2* 3’UTR restored reporter activity to control levels. Overexpression of miR‐193b significantly repressed *RRM2* expression at the mRNA and protein levels, and anti‐miR‐193b completely restored *RRM2* expression (Fig. 5B). These findings suggest that miR‐193b directly inhibits *RRM2* expression by targeting its 3’UTR.

We next assessed whether high expression of *RRM2* was correlated with clinical outcomes. In the Setlur cohort, higher levels of *RRM2* were significantly associated with increased Gleason score (*P* < 0.0001) and lethality (*P* = 0.0087, Fig. 5C). Metastatic cases of PC from the Yu and Lapotine cohorts (Lapointe *et al.*, 2004; Yu *et al.*, 2004) exhibited increased expression of *RRM2* (*P* < 0.0001, Fig. 5D). These data suggest that silencing of miR‐193b in PC could contribute to PC progression through derepression of *RRM2*.

### 3.6 Reduced DNA methylation restores expression of miR‐193b and its oncogenic targets

**Figure 6:**
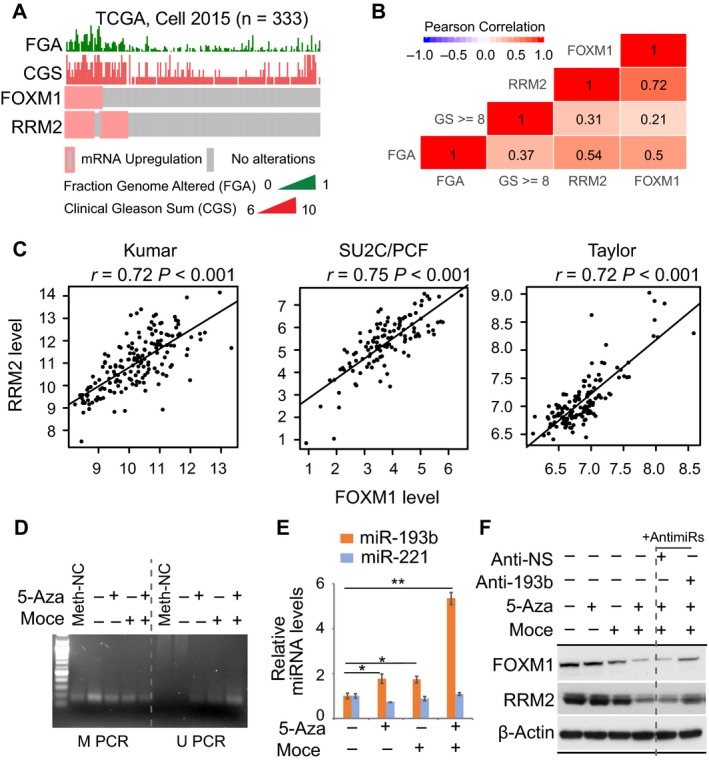
Reduced methylation of the miR‐193b promoter restores the inhibition of *FOXM1* and *RRM2*. (A) TCGA cases were assessed for mRNA upregulation of *FOXM1* and *RRM2*, Gleason sum, and fraction genome altered. Oncoprint generated by cBioPortal (Gao *et al.*, 2013). (B) Correlation plot shows significant (*P* < 0.05) Pearson correlation values. (C) The correlation between *FOXM1* and *RRM2* expression levels in 3 PC cohorts. (D) Changes in miR‐193b promoter methylation with drug treatments. Genomic DNA was isolated from the control and drug‐treated groups, and methylation status was evaluated by methylation‐specific PCR. M PCR, reaction specific for methylated miR‐193b promoter. U PCR, reaction specific for unmethylated miR‐193b promoter. Control reactions using methylated DNA are shown (Meth‐NC). (E) miR‐193b expression induced by 5‐Aza‐dC (5‐Aza) and mocetinostat (Moce). 22Rv1 cells were treated with 5‐Aza (5 µm) and Moce (1 µm) alone and in combination. miRNA levels were assessed after 24 h of treatment. (F) FOXM1 and RRM2 expression changes with drug treatments. 22Rv1 cells were transfected with anti‐miRNAs (nonspecific, A‐NS; anti‐miR‐193b, A‐193b) for 48 h and then treated with 5‐Aza‐dC and mocetinostat for 24 h. Values represent the mean ± SEM of three independent experiments. Statistical analysis was performed with one‐way ANOVA followed by Dunnett's test (for multiple group comparisons). *P < 0.05; **P < 0.01.

We and others have shown the clinical significance of both *FOXM1* and *RRM2* in PC (Aytes *et al.*, 2014; Ketola *et al.*, 2017; Mazzu *et al.*, 2019). Because both genes are regulated by miR‐193b, we investigated the relationships between *FOXM1* and *RRM2* expression and clinical attributes in PC samples. In TCGA, both *FOXM1* upregulation and *RRM2* upregulation are significantly correlated with high copy number alteration burden (indicated as fraction genome altered) and high Gleason score (Fig. 6A,B). There was a statistically significant correlation between *FOXM1* and *RRM2* levels in TCGA cohort (Fig. 6B), which we confirmed in 3 additional PC cohorts that include metastatic samples (Fig. 6C). These findings suggest that *FOXM1* and *RRM2* are jointly regulated and that methylation‐induced silencing of miR‐193b could contribute to the overexpression of *FOXM1* and *RRM2* and PC progression.

Because silencing of miR‐193b is mediated through hypermethylation of the miR‐193b promoter, we hypothesized that reducing DNA methylation would restore miR‐193b levels. The DNMT inhibitor (DNMTi) 5‐Aza‐2’‐deoxycytidine (5‐Aza‐dC) leads to decreased levels of DNA methylation and is used to treat hematologic malignancies (Nie *et al.*, 2014). Further, the combinatorial use of DNMTi with HDAC inhibitors (HDACi) can lead to the synergistic reactivation of silenced genes (Yang *et al.*, 2001). To examine whether decreased DNA methylation would restore expression of miR‐193b, we treated 22Rv1 cells with 5‐Aza‐dC and mocetinostat (a class I and IV HDACi) alone and in combination. We first evaluated how 5‐Aza‐dC and mocetinostat treatment altered the methylation state of the miR‐193b promoter by MSP. We observed a modest reduction in the methylation PCR product with treatment of either 5‐Aza‐dC or mocetinostat alone, and the combination treatment led to a marked reduction of the methylation PCR product (Fig. 6D). Correspondingly, unmethylated PCR products were only detected when cells were treated with 5‐Aza‐dC and mocetinostat alone or in combination (Fig. 6D). These results demonstrate that methylation of the miR‐193b promoter is decreased with treatment with DNMTi and HDACi.

Next, we examined whether decreased DNA methylation of the miR‐193b promoter altered expression of miR‐193b and its critical targets. Treatment of both 5‐Aza‐dC and mocetinostat alone induced a twofold increase in miR‐193b expression, and combination treatment increased miR‐193b expression by more than fivefold (Fig. 6E). Treatment with inhibitors did not alter the expression of the oncogenic miRNA miR‐221 (Sun *et al.*, 2009). We obtained similar results in LNCaP cells (Fig. S4A). Combination treatment also led to stronger inhibition of FOXM1 and RRM2 expression than treatment with either compound alone (Fig. 6F). Treatment with anti‐miR‐193b partially restored FOXM1 and RRM2 expression, further suggesting that miR‐193b inhibits expression of both proteins and that decreased miR‐193b promoter methylation can restore the function of miR‐193b. We also examined whether treatment with 5‐Aza‐dC and mocetinostat affected the viability of PC cells. There was no difference in the viability of LNCaP and 22Rv1 cells treated with 5‐Aza‐dC, and there was a 30% reduction in the viability of cells treated with mocetinostat (Fig. S4B). Intriguingly, combination treatment of 5‐Aza‐dC and mocetinostat significantly augmented the reduction of cell viability (Fig. S4B), suggesting a possible synergistic effect of the two inhibitors.

## 4 Discussion

Deregulation of miRNA has been reported in the early and late stages of PC (Daniel *et al.*, 2017; Massillo *et al.*, 2017; Ozen *et al.*, 2008; Schaefer *et al.*, 2010; Vanacore *et al.*, 2017), and aberrant epigenetic regulation may at least partially explain some miRNA expression alterations. Several miRNA (such as miR‐27a, miR‐141, miR‐200c, miR‐34a, and the miR‐130 family) have been shown to be hypermethylated in PC, resulting in the reduction or loss of their function (Barros‐Silva *et al.*, 2018; Kong *et al.*, 2012; Lynch *et al.*, 2016; Ramalho‐Carvalho *et al.*, 2017). Recent studies have revealed that miR‐193b is hypermethylated in PC tissues and in the urine of PC patients (Rauhala *et al.*, 2010; Torres‐Ferreira *et al.*, 2017), suggesting the potential for its future use as a biomarker. We confirmed the hypermethylation of miR‐193b in a larger patient cohort (TCGA, *n* = 333) and in multiple PC cell lines. However, we could not find a direct correlation between miR‐193b levels and Gleason score in TCGA cohort (data not shown). Because miRNA can regulate many targets, we hypothesized that the miR‐193b regulatory network might be correlated with clinical outcomes.

We nominated a set of 41 core genes as putative miR‐193b targets in PC cells using a published dataset of miR‐193b‐regulated genes in liposarcoma and gene expression analysis from PC subtypes. We observed significant inhibition of the majority of the 41 genes when miR‐193b was overexpressed in PC cells, indicating that a only small number are cell type‐specific targets of miR‐193b. High expression of these genes was associated with decreased survival of patients with PC, demonstrating that miR‐193b targets contribute to PC progression. We also revealed that approximately 50% of the genes that are activated in the PCS1 signature are miR‐193b targets; eleven of the 50 PAM50 genes in PC are also targets of miR‐193b (Fig. 3B). Both the PCS1 and PAM50 signatures have been reported to be associated with poor prognosis (You *et al.*, 2016; Zhao *et al.*, 2017), suggesting that decreased miR‐193b expression may be contributing to aggressive subtypes of PC.

Because miR‐193b overexpression induced distinct phenotypes in different PC cell lines, we hypothesize that the regulation of target gene expression by miR‐193b may be dependent on the basal expression level of the target. In 22Rv1 cells, miR‐193b overexpression induced modest apoptosis but significantly inhibited invasion. Similarly, knockdown of *FOXM1* significantly inhibited invasion of cells and led to decreased expression of *FOXM1* targets. In contrast, knockdown of *FOXM1* in LNCaP cells did not affect invasion and only modestly inhibited cell viability (Fig. S3). This suggests that *FOXM1* may not have a major role in tumorigenesis in LNCaP cells and that *RRM2* may be the primary target of miR‐193b. This is supported by our data demonstrating that LNCaP cells with either miR‐193b overexpression or *RRM2* knockdown had higher levels of apoptosis than control cells (Fig. 2B and Mazzu *et al.*, 2019). Based on these data, both *FOXM1* and *RRM2* may be the main targets of miR‐193b that contribute to PC metastases and decreased survival. Interestingly, both genes are in the PCS1 signature, and *FOXM1* has been reported as the master driver of the PCS1 subtype (Ketola *et al.*, 2017), further supporting this hypothesis.

Based on our data, we believe that hypermethylation of the miR‐193b promoter releases the inhibition of key PCS1 genes—including *FOXM1* and *RRM2*—and contributes to an aggressive phenotype. However, we could not detect a negative correlation between miR‐193b expression level and either *FOXM1* or *RRM2* expression levels in TCGA dataset (data not shown). Because the miR‐193b promoter is hypermethylated and expression levels in PC are low, it may be difficult to confirm a relationship *in vivo*. The positive correlation between *FOXM1* and *RRM2* expression in multiple cohorts may provide indirect support that both genes are jointly regulated by miR‐193b in PC. Our previous work demonstrated that FOXM1 transcriptionally activates *RRM2* in PC (Mazzu *et al.*, 2019), suggesting that transcriptional regulation may also contribute to the correlation between *FOXM1* and *RRM2* levels. Increased expression of miR‐193b targets *FOXM1* and *RRM2* is not only highly correlated with higher Gleason score, but also with an elevated fraction genome altered (FGA) signal. This is consistent with evidence demonstrating that increased FGA is correlated with higher Gleason score (Cancer Genome Atlas Research Network, 2015); elevated FGA is associated with genome instability, resulting in more genetic alterations and thereby contributing to PC progression. Interestingly, both FOXM1 and RRM2 have been found to play important roles in DNA repair and DNA integrity (D'Angiolella *et al.*, 2012; Nestal de Moraes *et al.*, 2016), which may explain their contribution to PC progression. As miRNA profiling in PC continues, we expect that additional data will facilitate the future discoveries about miR‐193b and its targets *in vivo*.

Because promoter methylation is a mechanism of miR‐193b silencing, we attempted to restore miR‐193b levels in PC cells using DNMTi and HDACi. Combination treatment had the most pronounced effect in decreasing miR‐193b promoter methylation, increasing miR‐193b expression, and reducing expression of miR1‐193b targets FOXM1 and RRM2. Although we believe that this reduced expression of FOXM1 and RRM2 is the result of decreased miR‐193b promoter methylation, it is possible that this is merely the result of a global decrease in DNA methylation and is independent of miR‐193b promoter methylation. DNA methylation and histone modifications are known to affect the localization of each other (Cedar and Bergman, 2009), and synergistic activation of some silenced gene promoters may require treatment with both DNMTi and HDACi (Cecconi *et al.*, 2009; Klisovic *et al.*, 2003; Walton *et al.*, 2008). Several clinical trials have demonstrated complete and partial remissions of hematologic and solid tumors in patients treated with combination DNMTi and HDACi (Griffiths and Gore, 2008; Rudek *et al.*, 2005). Our results suggest that a similar regimen may be a future therapeutic strategy for patients with PC who have a hypermethylated miR‐193b promoter.

## 5 Conclusions

Hypermethylation of the miR‐193b promoter may contribute to the progression of aggressive PC. We identified a set of target genes that are inhibited by miR‐193b and whose activation is associated with decreased survival in human PC. We have shown that deregulation of critical miR‐193b targets *FOXM1* and *RRM2* alters the tumorigenic nature of PC cell lines and is associated with more aggressive phenotypes. Treatment with DNMTi and HDACi restores miR‐193b expression and the inhibition of *FOXM1* and *RRM2* and may be a future strategy for treatment of patients with PC who harbor a hypermethylated miR‐193b promoter.

## Conflict of interest

As of February 18, 2019, P.W. Kantoff reports the following disclosures for the last 36‐month period: He has investment interest in Context Therapeutics LLC, DRGT, Placon, Seer Biosciences, and Tarveda Therapeutics; he is a company board member for Context Therapeutics LLC; he is a consultant/scientific advisory board member for BIND Biosciences, Inc., Bavarian Nordic Immunotherapeutics, DRGT, GE Healthcare, Janssen, Metamark, New England Research Institutes, Inc., OncoCellMDX, Progenity, Sanofi, Seer Biosciences, Tarveda Therapeutics, and Thermo Fisher; and he serves on data safety monitoring boards for Genentech/Roche and Merck. All other authors have no potential conflicts to disclose.

## Author contributions

YZM and PWK performed the conception and design. YZM involved in the development of methodology. YZM, GC, YY, SN, JA, GSL, and LJ involved in the acquisition of data. YZM, SN, and JA involved in the analysis and interpretation of data. YZM, SN, and PWK wrote, reviewed, and revised the manuscript. YZM, YY, and SN performed the administrative, technical, or material support. PWK involved in the study supervision.

## Supporting information


**Fig. S1.** Tumor suppressive properties of miR‐193b in PC cells. A, decrease in cell viability with miR‐193b overexpression in PC cell lines. Cell viability was assessed in multiple miRNA‐transfected PC cells. All values were normalized to the nonspecific control miRNA (NS) value on Day 0. B, miR‐193b overexpression in PC cell lines. Values were normalized to NS cells. C, left, increased cell viability of LAPC4 cells treated with the miR‐193b inhibitor anti‐miR‐193b (A‐193b) relative to cells treated with the nonspecific (A‐NS) control. Right, decreased miR‐193b expression in LAPC4 cells treated with A‐193b. D, modest apoptosis induced by miR‐193b overexpression in 22Rv1 cells. Left, FACS plots with percentages of cells in each quadrant shown. Right, quantification of FACS results. E, inhibition of cell invasion by miR‐193b overexpression in PC‐3 cells. Left, representative image of results. Right, quantification of results. Scale bar: 200 µm. Values represent the mean ± SEM of 3 independent experiments. Student's t‐test was used for p value calculation. *, p<0.05; **, p<0.01; ***, p<0.001.

**Fig. S2.** miR‐193b regulation of 41 PCS1 genes in PC cells. A, miRNA with the highest potential of targeting the 41 genes. Toppgene analysis was used to predict miRNA targeting the 41 genes, and the top 5 miRNA are listed. B, inhibition of 41 genes by miR‐193b overexpression in PC‐3 cells. Gene expression was evaluated by qRT‐PCR in miR‐193b‐transfected PC‐3 cells, and values were normalized to cells transfected with a nonspecific control miRNA. C, miR‐193b expression levels in 3 PC cell lines 72 hours after miR‐193b transfection. Value were normalized to the control group (NS) for each cell line. D, pathway enrichment of 41 genes inhibited by miR‐193b in PC. Values represent the mean ± SEM of 3 independent experiments.

**Fig. S3.** Knockdown of *FOXM1* in PC cells. A, FOXM1 and RRM2 protein levels in LNCaP and 22Rv1 cells. B, inhibition of cell viability by si*FOXM1* in LNCaP cells. C, cell invasion assay in LNCaP cells transfected with nonspecific siRNA (siNS) and siRNA targeting *FOXM1 *(siFOXM1). Left, representative image of results. Right, quantification of results. Scale bar: 100 µm. D, FOXM1 level in siRNA‐transfected 22Rv1 and LNCaP cells. Values represent the mean ± SEM of 3 independent experiments. Student's t‐test was used for p value calculation. *, p<0.05; ns: not significant.

**Fig. S4.** Treatment of PC cells with 5‐Aza‐dC and mocetinostat. A, miR‐193b expression induced by 5‐Aza‐dC and mocetinostat. LNCaP cells were treated with 5 µM 5‐Aza‐dC (5‐Aza) and 1 µM mocetinostat (Moce) alone or in combination for 24 hours, and miRNA levels were assessed. B, assessment of cell viability after treatment with 5‐Aza‐dC and mocetinostat. LNcaP and 22Rv1 cells were treated with 5 µM 5‐Aza and 1 µM Moce alone or in combination for 72 hours, and cell viability was assessed. The values represent the mean ± SEM of 3 independent experiments. Student's t‐test was used for p value calculation. *, p<0.05; **, p<0.01.

**Table S1.** Probe and primer information.

**Table S2.** Primer information for the PrimePCR Assays.

**Table S3.** Summary of prostate cancer clinical cohorts.
