# Supplementary figures and images for: Methylation‐associated miR‐193b silencing activates master drivers of aggressive prostate cancer
Source: Mol Oncol. 2019 Jul 19;13(9):1944–58. doi: 10.1002/1878-0261.12536 (PMC6717747; doi:10.1002/1878-0261.12536)

Figure S1

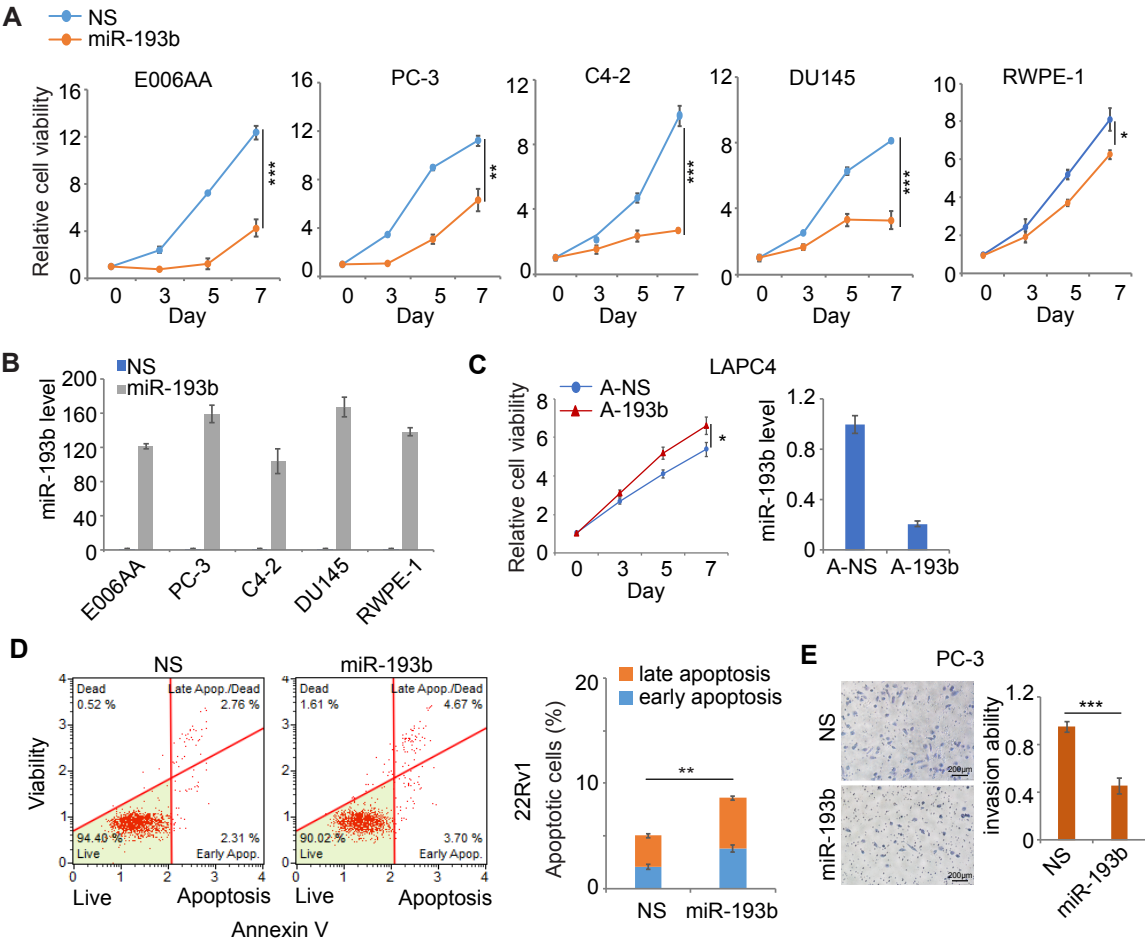

Supplement: Supplementary file 1 — Fig. S1. Tumor suppressive properties of miR‐193b in PC cells. A, decrease in cell viability with miR‐193b overexpression in PC cell lines. Cell viability was assessed in multiple miRNA‐transfected PC cells. All values were normalized to the nonspecific control miRNA (NS) value on Day 0. B, miR‐193b overexpression in PC cell lines. Values were normalized to NS cells. C, left, increased cell viability of LAPC4 cells treated with the miR‐193b inhibitor anti‐miR‐193b (A‐193b) relative to cells treated with the nonspecific (A‐NS) control. Right, decreased miR‐193b expression in LAPC4 cells treated with A‐193b. D, modest apoptosis induced by miR‐193b overexpression in 22Rv1 cells. Left, FACS plots with percentages of cells in each quadrant shown. Right, quantification of FACS results. E, inhibition of cell invasion by miR‐193b overexpression in PC‐3 cells. Left, representative image of results. Right, quantification of results. Scale bar: 200 µm. Values represent the mean ± SEM of 3 independent experiments. Student's t‐test was used for p value calculation. *, p<0.05; **, p<0.01; ***, p<0.001. [file MOL2-13-1944-s001.pdf]

Figure S2

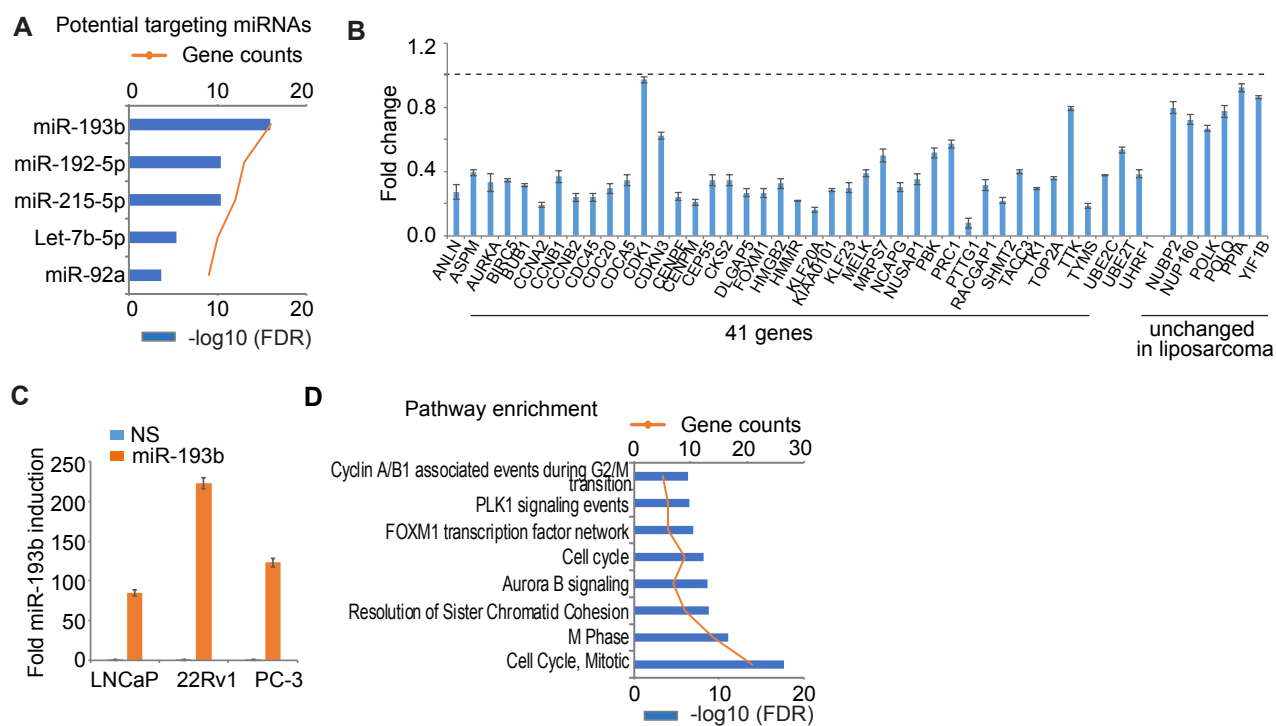

Supplement: Supplementary file 2 — Fig. S2. miR‐193b regulation of 41 PCS1 genes in PC cells. A, miRNA with the highest potential of targeting the 41 genes. Toppgene analysis was used to predict miRNA targeting the 41 genes, and the top 5 miRNA are listed. B, inhibition of 41 genes by miR‐193b overexpression in PC‐3 cells. Gene expression was evaluated by qRT‐PCR in miR‐193b‐transfected PC‐3 cells, and values were normalized to cells transfected with a nonspecific control miRNA. C, miR‐193b expression levels in 3 PC cell lines 72 hours after miR‐193b transfection. Value were normalized to the control group (NS) for each cell line. D, pathway enrichment of 41 genes inhibited by miR‐193b in PC. Values represent the mean ± SEM of 3 independent experiments. [file MOL2-13-1944-s002.pdf]

Figure S3

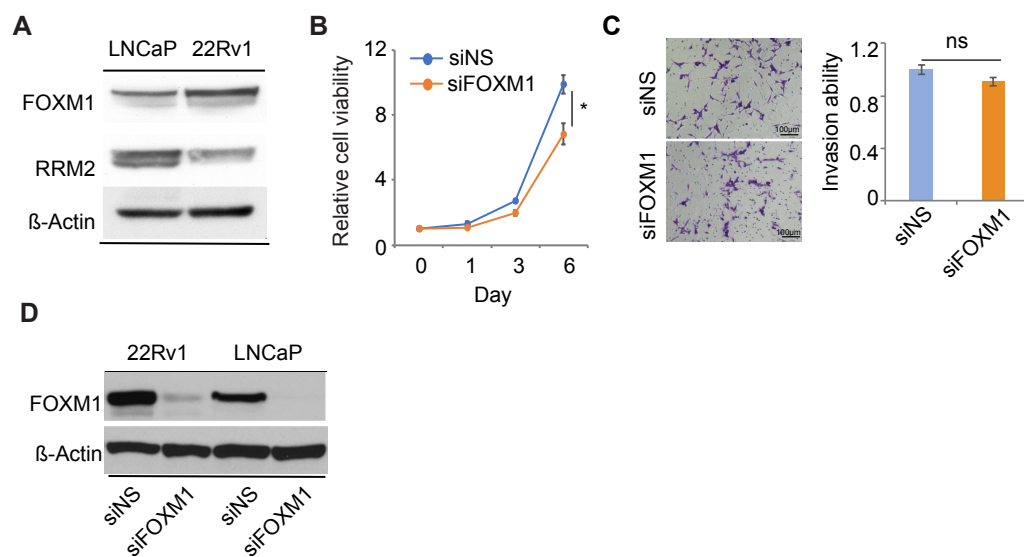

Supplement: Supplementary file 3 — Fig. S3. Knockdown of FOXM1 in PC cells. A, FOXM1 and RRM2 protein levels in LNCaP and 22Rv1 cells. B, inhibition of cell viability by siFOXM1 in LNCaP cells. C, cell invasion assay in LNCaP cells transfected with nonspecific siRNA (siNS) and siRNA targeting FOXM1 (siFOXM1). Left, representative image of results. Right, quantification of results. Scale bar: 100 µm. D, FOXM1 level in siRNA‐transfected 22Rv1 and LNCaP cells. Values represent the mean ± SEM of 3 independent experiments. Student's t‐test was used for p value calculation. *, p<0.05; ns: not significant. [file MOL2-13-1944-s003.pdf]

Figure S4

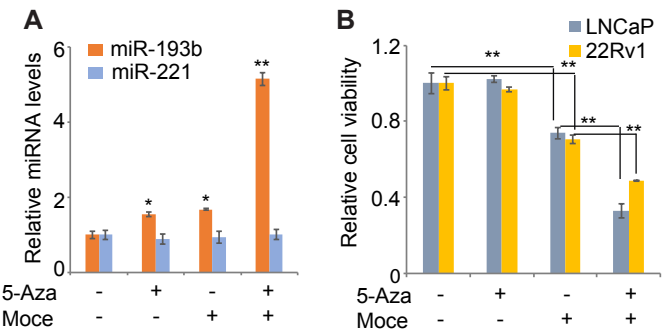

Supplement: Supplementary file 4 — Fig. S4. Treatment of PC cells with 5‐Aza‐dC and mocetinostat. A, miR‐193b expression induced by 5‐Aza‐dC and mocetinostat. LNCaP cells were treated with 5 µM 5‐Aza‐dC (5‐Aza) and 1 µM mocetinostat (Moce) alone or in combination for 24 hours, and miRNA levels were assessed. B, assessment of cell viability after treatment with 5‐Aza‐dC and mocetinostat. LNcaP and 22Rv1 cells were treated with 5 µM 5‐Aza and 1 µM Moce alone or in combination for 72 hours, and cell viability was assessed. The values represent the mean ± SEM of 3 independent experiments. Student's t‐test was used for p value calculation. *, p<0.05; **, p<0.01. [file MOL2-13-1944-s004.pdf]
