# Supplementary material for: Methylation‐associated miR‐193b silencing activates master drivers of aggressive prostate cancer
Source: Mol Oncol. 2019 Jul 19;13(9):1944–58. doi: 10.1002/1878-0261.12536 (PMC6717747; doi:10.1002/1878-0261.12536)
Supplement: Supplementary file 5 — Table S1. Probe and primer information. [file MOL2-13-1944-s005.docx]

**Table S1 Probe and primer information**

| **TaqMan probe** | **Name** | **Assay ID** |
| --- | --- | --- |
|  | CCNA2 | Hs00996788_m1 |
|  | CDK1 | Hs00938777_m1 |
|  | BIRC5 | Hs04194392_s1 |
|  | CCNB1 | Hs01030099_m1 |
|  | CCNB2 | Hs01084593_g1 |
|  | CENPF | Hs01118845_m1 |
|  | FOXM1 | Hs01073586_m1 |
|  | GAPDH | Hs02786624_g1 |
|  | RRM2 | Hs00357247_g1 |
| **miRNA primer** | **Name** | **Sequence** |
|  | miR-193b | AACTGGCCCTCAAAGTCCCGCT |
|  | miR-221 | AGCTACATTGTCTGCTGGGTTTC |
| **MSP primer** | **Name** | **Sequence** |
|  | M1636-For | ATTGTACGAGGTATTTCGGATATTC |
|  | M1636-Rew | CCATAACTAAAAACAAAAAAATCGC |
|  | U1636-For | TGTATGAGGTATTTTGGATATTTGA |
|  | U1636-Rew | CCATAACTAAAAACAAAAAAATCACT |
