# Supplementary material for: Methylation‐associated miR‐193b silencing activates master drivers of aggressive prostate cancer
Source: Mol Oncol. 2019 Jul 19;13(9):1944–58. doi: 10.1002/1878-0261.12536 (PMC6717747; doi:10.1002/1878-0261.12536)
Supplement: Supplementary file 7 — Table S3. Summary of prostate cancer clinical cohorts. [file MOL2-13-1944-s007.docx]

| **Cohort name** | **Benign/normal tissue number** | **Tumor number** | **Primary number** | **Metastasis number** | **Clinical outcome** | **Data type** | **Year** | **Journal** | **Reference** |
| --- | --- | --- | --- | --- | --- | --- | --- | --- | --- |
| TCGA | 0 | 333 | 333 | 0 | Biochemical recurrence | Whole-exome seq, RNA seq, RPPA | 2015 | Cell | 1, 2 |
| Taylor | 29 normal | 216 | 131 | 19 | Biochemical recurrence | aCGH, RNA-seq | 2010 | Cancer Cell | 3 |
| SU2C/PCF | 0 | 150 | 0 | 150 | Biochemical recurrence | Whole-exome seq, RNA seq | 2015 | Cell | 4 |
| Kumar | 176 benign | 176 | 22 | 154 | Biochemical recurrence | aCGH, whole-exome seq, microarray | 2016 | Nat Med | 5 |
| Grasso | 29 benign | 94 | 59 | 35 | Overall survival | aCGH, microarray | 2012 | Nature | 6 |
| Yu | 23 normal | 89 | 64 | 25 | Survival status | Microarray | 2004 | J Clin Oncol | 7 |
| LaTulippe | 3 normal | 32 | 23 | 9 | Biochemical recurrence | Microarray | 2002 | Cancer Res | 8 |
| Lapointe | 41 normal | 71 | 62 | 9 | Biochemical recurrence, survival | Microarray | 2004 | PNAS | 9 |
| Setlur | 0 | 363 | 363 | 0 | Survival status | Microarray | 2008 | J Natl Cancer Inst | 10 |
| Varambally | 6 benign | 13 | 7 | 6 | N/A | Microarray | 2005 | Cancer Cell | 11 |

aCGH, array comparative genomic hybridization; RPPA, reverse phase protein array

**References**

1. Cancer Genome Atlas Research Network. The Molecular Taxonomy of Primary Prostate Cancer. *Cell* 2015; **163**: 1011–1025.

2. TCGA Data Portal: https://tcga-data.nci.nih.gov/docs/publications/tcga

3. Taylor BS, Schultz N, Hieronymus H, Gopalan A, Xiao Y, Carver BS *et al*. Integrative genomic profiling of human prostate cancer. *Cancer Cell* 2010; **18**: 11–22.

4. Robinson D, Van Allen EM, Wu YM, Schultz N, Lonigro RJ, Mosquera JM *et al*. Integrative clinical genomics of advanced prostate cancer. *Cell* 2015; **161**: 1215–1228.

5. Kumar A, Coleman I, Morrissey C, Zhang X, True LD, Gulati R *et al*. Substantial interindividual and limited intraindividual genomic diversity among tumors from men with metastatic prostate cancer. *Nat Med* 2016; **22**: 369–378.

6. Grasso CS, Wu YM, Robinson DR, Cao X, Dhanasekaran SM, Khan AP *et al*. The mutational landscape of lethal castration-resistant prostate cancer. *Nature* 2012; **487**: 239–243.

7. Yu YP, Landsittel D, Jing L, Nelson J, Ren B, Liu L et al. Gene expression alterations in prostate cancer predicting tumor aggression and preceding development of malignancy. *J Clin Oncol* 2004; **22**: 2790–2799.

8. LaTulippe E, Satagopan J, Smith A, Scher H, Scardino P, Reuter V et al. Comprehensive gene expression analysis of prostate cancer reveals distinct transcriptional programs associated with metastatic disease. *Cancer Res* 2002; **62**:4499–4506.

9. Lapointe J, Li C, Higgins JP, van de Rijn M, Bair E, Montgomery K et al. Gene expression profiling identifies clinically relevant subtypes of prostate cancer. *Proc Natl Acad Sci U S A* 2004; **101**: 811–816.

10. Setlur SR, Mertz KD, Hoshida Y, Demichelis F, Lupien M, Perner S et al. Estrogen-dependent signaling in a molecularly distinct subclass of aggressive prostate cancer. *J Natl Cancer Inst* 2008; **100**: 815–825.

11. Varambally S, Yu J, Laxman B, Rhodes DR, Mehra R, Tomlins SA et al. Integrative genomic and proteomic analysis of prostate cancer reveals signatures of metastatic progression. *Cancer Cell* 2005; **8**: 393–406.
